# Using Loss of Balance to Understand Postural Instability Associated With Parkinson’s Disease

**DOI:** 10.1155/padi/6998888

**Published:** 2026-08-26

**Authors:** Ryan D. Kaya, Andrew Bazyk, Colin Waltz, Eric Zimmerman, Joshua D. Johnston, Benjamin L. Walter, Adam Margolius, Anson B. Rosenfeldt, Mandy Miller Koop, Jay L. Alberts

**Affiliations:** ^1^ Center for Neurological Restoration, Neurological Institute, Cleveland Clinic, Cleveland, Ohio, USA, clevelandclinic.org; ^2^ Department of Biomedical Engineering, Research Institute, Cleveland Clinic, Cleveland, Ohio, USA, clevelandclinic.org

## Abstract

**Background/Objectives:**

Postural instability compromises balance, contributing to annual fall rates of 45%–68% in Parkinson’s disease (PD). A fundamental gap in clinical evaluation and treatment of postural instability is the use of insufficiently challenging postural control tests and reliance on subjective scoring. Traditional 3D motion capture (Traditional‐MC), while precise and objective, is not feasible for clinical utilization. Markerless motion capture (MMC) is a viable candidate for quantifying postural control, feasible with embedded cameras of augmented reality (AR) headsets. This project aimed to assess MMC accuracy in quantifying postural control and to compare PD patients and healthy controls (HCs) to develop a loss‐of‐balance (LOB) prediction model.

**Methods:**

Video data were acquired by a clinician wearing a Microsoft HoloLens 2 AR headset as participants completed three progressively challenging postural control stances. Depth and red–green–blue camera data were analyzed with our custom‐built human pose estimation software (CART‐MMC). Criterion validity between Traditional‐MC and CART‐MMC was completed on outcomes, 95% ellipsoid sway area (Sway area) and 95% mediolateral and anteroposterior (ML and AP) ranges in 54 (HC = 28, PD = 26) participants. Group differences were assessed, and survival analysis was conducted to quantify no LOB probabilities in 31 HCs and 68 PD patients. A relaxed LASSO model was used to predict LOB in the PD group from CART‐MMC data.

**Results:**

Sway area and ML range from CART‐MMC were equivalent to Traditional‐MC. Furthermore, ML range from CART‐MMC differentiated PD patients from HCs in the stance that had the highest completion rate. As postural task difficulty increased, fewer PD patients were able to complete compared to HCs, resulting in lower survival probabilities of the PD group in Tandem‐Firm‐EO and FT‐Foam‐EC stances. In the least challenging stance, ML range predicted the occurrence of LOB in the more challenging stance (LOO‐CV AUC = 0.78).

**Conclusions:**

CART‐MMC is a valid method of obtaining objective postural control outcomes, which can detect latent postural control deficits and demonstrates a scalable, objective clinical and remote monitoring approach for predicting falls.

## 1. Introduction

Impaired postural control in Parkinson’s disease (PD) negatively affects functional mobility and independence with activities of daily living and often leads to social isolation due to fear of falling [1]. Fear of falling in people with Parkinson’s disease (PwPD) is well‐founded, as annual fall rates are more than three times higher than in healthy controls (HCs) [2]. Despite the high risk of falls, clinical balance assessments to stratify fall risk and balance deficits rely on scoring that categorizes performance into binary outcomes of completion (i.e., yes or no), time holding a stance, and subjectively rated numerical categorization of performance while ignoring the nuances of postural control [3, 4]. Inaccurate classification of low fall risk and balance impairment level can be concluded from clinical assessments due to a tendency of a ceiling effect, when in fact postural control deficits exist [3]. Although valued for the ability to be administered quickly and with minimal investment in equipment and training, clinical balance assessments have the propensity to be administered and scored with variability, supporting a need for objective quantification of postural control [5].

Posturography, including force plates, inertial measurement units, and marker‐based motion capture, has the precision to measure subtle, but important declines in postural control across the continuum of PwPD [6]. Sway detection in specific planes of movement provides refined insights into postural instability, as larger excursions along the mediolateral (ML) axis are a more sensitive measure of impaired postural control compared to total sway area [7] and are associated with recurrent fallers and prospective fall prediction in PD and older adults [8]. Thus, detection of postural instability using tests that challenge postural control while using objective biomechanical methods has potential to reduce falls and improve quality of life in PwPD [9].

Markerless motion capture (MMC) technology is an emerging approach that offers a low‐cost option to quantify the kinematics underlying postural control. In contrast to expensive, time‐ and space‐consuming marker motion capture systems and posturography technology, MMC is comprised of a camera system and human pose estimation software to provide cost‐effective and portable biomechanical movement analyses ideal for clinical use [10]. The Comprehensive Augmented Reality Testing (CART) platform [11, 12], designed for the quantification of PD symptoms, leverages MMC to acquire objective and quantifiable outcomes derived from clinician‐ and patient‐worn head‐mounted augmented reality headsets. The CART platform provides equivalent measures of upper‐extremity fine motor function (e.g., finger‐tapping) [11] and lower extremity performance (e.g., stepping in place) relative to a gold‐standard 3D motion capture system [12]. Over the past decade, multiple forms of MMC technology have been used to measure postural control in young adults and neurologically impaired populations [13]. While these studies suggest initial promise, they have not systematically evaluated MMC systems in the recommended rigorous manner [14]. Equivalence testing is the preferred method to ensure rigorous validation as it determines if a newly developed system is accurately measuring movement relative to a gold‐standard system [15]. Using the equivalence analysis framework from previous CART platform publications, postural control metrics from the CART platform were rigorously validated to ensure trustworthy postural control data could be used in research and clinical environments [16].

MMC technology is scalable and can potentially be deployed across clinical and home settings. Home‐based assessments have the potential to revolutionize neurological care by expanding specialty care to underserved areas. However, safety concerns, especially fall risk when evaluating standing postural control, should be considered. In HCs, posturography measures of ML excursion have predicted fallers versus nonfallers [17]. With the objective CART‐MMC data, postural control outcomes, such as ML excursion, can be used to predict loss of balance (LOB) in more challenging stances. Acquisition of LOB data may eliminate the need for attempting to assess highly challenging and potentially unsafe stances, especially when considering the remote, at‐home assessment.

The primary aim of this project was to assess criterion validity of the CART‐MMC against traditional motion capture (Traditional‐MC) for measuring postural control. It was hypothesized that the CART‐MMC would be within the 5% equivalence bounds for tracking postural control. The secondary aim was to exhibit known‐group validity by demonstrating differences in postural control and LOB outcomes between the HC and the PD groups. It was hypothesized that the PD group would exhibit increased COM sway and increased LOB episodes compared to the HC group. An exploratory aim was to develop a prediction model for detecting LOB during challenging, failure‐prone stances.

## 2. Methods

### 2.1. Participants

People with PD and age‐matched HCs participated in the study. Inclusion criteria for the PD group were (1) diagnosis of idiopathic PD, (2) Hoehn and Yahr Stage I–IV [18], (3) absence of other neurological conditions, (4) ability to follow 2‐step commands, and (5) stable anti‐Parkinsonian medication regimen for a minimum of 1 month prior to assessment. Inclusion criteria for the HC group were (1) absence of any neurological condition and (2) ability to follow 2‐step commands. For both groups, exclusion criteria were (1) implanted deep brain stimulation device, (2) history of a gait‐altering musculoskeletal injury, or (3) uncorrected vision or hearing impairments that would impact interaction with an AR headset. The study was approved by the Cleveland Clinic Institutional Review Board, and all participants provided informed consent prior to data collection.

For PwPD, data were collected off‐medication, defined as a minimum of 12 h after the participant’s last anti‐Parkinsonian medication dose. Motor symptom severity was assessed with the MDS‐UPDRS III by an MDS‐certified examiner, and disease severity was classified as mild, moderate, or severe (mild = MDS‐UPDRS III score of 0–32, moderate = 33–58, and severe = 59+) [19].

### 2.2. Postural Control Assessments: Setup and Procedure

**FIGURE 1:**
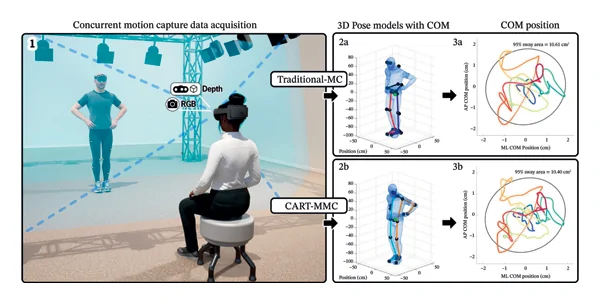
Experimental setup of the CART postural control task (FT‐Firm‐EC) collected from the Traditional‐MC and CART‐MMC systems visualized in the left panel (Panel 1). With standardized instructions from the participant‐worn HL2 device, the participant performed three balance stances. Images used for CART‐MMC were captured from a clinician‐worn HL2 (Panel 2b) simultaneously with Traditional‐MC recording active marker positions (Panel 2a). Traditional‐MC and CART‐MMC performed weighted segment averaging to construct an estimated COM point for each data stream (green dots, Panels 2a and 2b). The estimated COM points were compared for each data stream per postural control stance to assess equivalence (Panels 3a and 3b). Representative sway areas demonstrate the accuracy in capturing postural control data in CART‐MMC against Traditional‐MC.

Participants completed three postural control assessments for 30 s each while wearing the Microsoft HoloLens 2 (Microsoft Corporation, Redmond, WA; HL2) headset. Figure 1 provides a representation of the data collection and data processing for Traditional‐MC and CART‐MMC. Postural control assessments included (1) standing with feet together on a firm surface with eyes closed (FT‐Firm‐EC), (2) standing in tandem on a firm surface with eyes open (Tandem‐Firm‐EO), and (3) standing with feet together on a foam surface with eyes closed (FT‐Foam‐EC). The foam surface was a balance pad commonly used in rehabilitation settings (Airex AG, Sins, Switzerland). Standardized task instructions were delivered by a human‐like avatar that provided a visual demonstration with accompanying auditory instructions. Participants were instructed to maintain their hands on their hips during the 30‐s trials and to take a recovery step should they lose their balance. A spotter was available as needed for safety.

The clinician, wearing an HL2, was seated ∼10 feet in front of and facing the participant. The clinician’s HL2 captured standard red–green–blue (RGB) images, depth images, and camera parameters used for CART‐MMC.

To evaluate criterion validity, 54 participants’ (28 HC, 26 PD) CART‐MMC data were compared against Traditional‐MC (Figure 1). For Traditional‐MC, 19 LED active markers (SuperNova; Vicon Motion Systems, Oxford, UK) were placed at anatomical landmarks defined by the modified Plug‐in‐Gait marker setup [20], which have been previously published [12]. The active markers were powered by a battery pack that was placed in a waist‐worn holster. Traditional‐MC recorded the active marker positions from 16 motion capture cameras (Vicon Motion Systems, Oxford, UK) at 100 Hz. Trial start and stop were synchronized between the HL2 and Traditional‐MC via triggers sent over a local network.

### 2.3. CART‐MMC Human Pose Model

The CART HL2 application was developed in Unity 2021.3.4f1 using C#, Microsoft Mixed Reality Toolkit 3.0.0‐pre14, Microsoft Mixed Reality OpenXR Plugin 1.8.0, and Unity OpenXR Plugin 1.6.0. The HL2 utilized the Windows Holographic operating system Version 23H2, build number: 22621.1258.

To collect HL2 camera images and camera parameters from the clinician’s headset for the CART‐MMC method, Microsoft’s Research Mode was used. A dynamic‐link library plugin written in C++ used the Research Mode API to obtain camera‐related data. RGB images were collected at 30 Hz with a resolution of 1280 × 720, and depth long‐throw images were sampled at 5 Hz with a resolution of 320 × 288. RGB and depth camera parameters were saved to text files.

The CART‐MMC analysis was performed offline in MATLAB (MathWorks, MATLAB R2022b) as previously published [12]. Briefly, the RGB images from the clinician’s headset were tracked using Google MediaPipe pose landmark detection software (Version 0.10.13). Thirty‐three pose landmark positions were identified for each RGB image. Each depth image acquired from the clinician headset was temporally matched to the closest RGB image, and the combination was used to calculate an RGB‐depth composite image. For RGB images without paired depth images, depth values for each pose landmark were linearly interpolated from depth values determined from RGB‐depth composite images. Following depth interpolation, every RGB frame contained RGB image coordinates and corresponding depth values for each of the 33 pose landmarks. The three‐dimensional world pose landmarks were computed for CART‐MMC, resulting in a human pose model of 3D positions of 33 pose landmarks at 30 Hz.

### 2.4. Traditional‐MC Human Pose Model

Traditional‐MC data were processed with Vicon software (Nexus 2.16.0). The raw marker positions of the 19 markers were reconstructed and labeled for each trial. Data integrity was confirmed and processed with a custom Nexus pipeline. The final pose model contained 3D marker positions and calculated joint center positions.

### 2.5. Data Processing and Metric Calculations

#### 2.5.1. Signal Preprocessing

All CART‐MMC and Traditional‐MC pose landmark positions were resampled to 60 Hz using linear interpolation. The first and last 1 s of each trial were removed. Data for each pose landmark were then filtered using a fourth‐order Butterworth lowpass filter with a 3‐Hz cutoff frequency. Due to a common vertical axis between both systems, determined from calibration for Traditional‐MC and spatial mapping for CART‐MMC, spatial alignment was only necessary on the anteroposterior (AP) and ML axes. To spatially align the two systems, the average vector pointing from left to right hips across the entire trial was aligned to the *x*‐axis. Specifically, the left and right hip joint centers were aligned to the *x*‐axis for Traditional‐MC and the MediaPipe‐defined hip positions were aligned to the *x*‐axis for CART‐MMC.

#### 2.5.2. Calculation of Center of Mass

For each system, COM was calculated using the weighted averaging of body segments described by de Leva [21] using a lower limb and trunk model [22]. Sex‐specific longitudinal positions were used for male and female participants.

##### 2.5.2.1. CART‐MMC

Body segments used for the CART‐MMC COM calculation were the trunk, thighs, shanks, and feet. MediaPipe pose landmarks were used to define the body segments. Trunk endpoints were the midpoint of the shoulders to the midpoint of the hips, thigh endpoints were the hip joint to the knee joint, shank endpoints were the knee joint to the ankle joint, and foot endpoints were the heel to the distal hallux.

##### 2.5.2.2. Traditional‐MC

Body segments used for the Traditional‐MC COM calculation were the trunk, thighs, shanks, and feet. Trunk endpoints were cervical vertebrae 7 to the midhip, thigh endpoints were the hip joint center to the knee joint center, shank endpoints were the knee joint center to the ankle joint center, and foot endpoints were the heel to the distal hallux.

#### 2.5.3. Spatiotemporal Alignment Between Traditional‐MC and CART‐MMC

Following spatial alignment, COM data were used to temporally align the poses by minimizing the root mean squared deviation between COM across temporal shifts. Excess data shifted to the start or end of the trial due to temporal alignment were removed to guarantee both poses had the same number of samples aligned in time.

#### 2.5.4. Automated LOB Detection

LOB detection was automated with a custom algorithm written in MATLAB. Both LOB as a binary measure and the time at which LOB occurred were detected. A LOB was defined as a step that exceeded a simultaneous excursion of the CART‐MMC‐defined knee and ankle greater than or equal to 4 and 8 cm, respectively. The value of 4 cm at the knee was utilized as it is 2–3× the noise floor of CART‐MMC. Thresholding above the noise floor minimized the influence of the model noise on falsely detecting an LOB. The value of 8 cm at the ankle was utilized based on the anatomy and biomechanics of the lower extremity; a compensatory step would require an excursion at the ankle twice the distance of the knee, assuming the knee is at an equal distance between the CART‐MMC‐defined hip and ankle joints.

#### 2.5.5. Postural Control Metric Calculations

Time domain metrics to characterize postural control were calculated for each stance with a custom MATLAB program for Traditional‐MC and CART‐MMC. Metrics calculated included 95% range of the ML movements (ML range), 95% range of the AP movements (AP range), and 95% ellipsoid sway area (Sway area) [23]. For ML and AP ranges, data are expressed in centimeters (cm), and Sway area is presented in cm^2^. 95% bounds were selected for ML and AP ranges and way area to represent typical postural control while limiting the effect of outliers in the trial data.

### 2.6. Statistical Analysis

Demographic metrics were calculated using appropriate summary statistics. As postural control metrics were right‐skewed, these metrics were log‐transformed to normality. As metrics were approximately log‐normally distributed, the back‐transformed means represent participant medians. All statistical analyses were conducted using RStudio 2025.09.0, R Version 4.5.1.

#### 2.6.1. Criterion Validity Statistical Analysis

Agreement in postural control metrics between the Traditional‐MC and CART‐MMC systems was assessed using linear mixed models (LMMs) to test equivalence between systems within each stance across PD and HC combined who were able to complete their assessment without an LOB. For each outcome, an LMM was constructed on data across all three stances with fixed effect terms for stance, system, and the interaction of stance and system, and random intercepts by participant and trial nested within participant. If there was a significant interaction of stance and system (i.e., if agreement between systems depended on stance), equivalence was tested between systems separately within each stance. If a significant interaction was not observed, equivalence between systems was tested across all stances simultaneously. Equivalence testing was conducted by testing if the estimated difference between systems was significantly within the a priori allowable equivalence range of ±5%.

#### 2.6.2. Known‐Group Validity Statistical Analysis

Differences by group in postural control outcomes were assessed using Welch’s *t*‐test within each stance for participants who were able to complete their assessment without an LOB.

#### 2.6.3. LOB Detection Analysis

The accuracy, sensitivity, and specificity of the CART‐MMC’s LOB detection algorithm were quantified against clinician‐identified LOB detection, collapsing trial stance. Offline, a clinician independently reviewed all trials for LOB, which was defined as at least one foot losing contact with the stance surface. The time at which the clinician observed an LOB was recorded. For trials able to be started and containing a LOB, as determined by both the CART‐MMC and the clinician, the agreement in time (seconds) until LOB was identified using a LMM predicting time until LOB with a fixed term for detection method (CART‐MMC or clinician) and random intercepts by trial nested within participant. Equivalence of the mean time until LOB from the CART‐MMC’s automated LOB detection algorithm against the mean clinician time until LOB was tested using the LMM with equivalence bounds of ±5%.

#### 2.6.4. Kaplan–Meier Survival Curves and Log‐Rank Analysis

Group differences in the ability to complete were assessed by comparing time without an LOB as measured by the CART‐MMC detection algorithm via log‐rank tests within each stance. Participants who were unable to start the assessment due to an inability to assume the initial position were assigned a time until LOB of 0 s. Time without an LOB was visually depicted via Kaplan–Meier curves.

#### 2.6.5. Predicting LOB in the Tandem‐Firm‐EO Stance

LOB in the Tandem‐Firm‐EO as determined by the CART‐MMC was predicted using outcome data from the FT‐Firm‐EC by building a relaxed least absolute shrinkage and selection operator (LASSO) logistic regression model. As the outcomes may be highly correlated, LASSO regularization was used to potentially select only one outcome and avoid multicollinearity. The regularization process was allowed to be relaxed, meaning shrinkage was used to determine which outcomes to be included in the model, but coefficients were then refit without penalization. The lambda parameter was determined via 10‐fold cross‐validation. The model performance was assessed using leave‐one‐out cross‐validation (LOO‐CV), in which the entire modeling process was repeatedly done, leaving a single point out, then parameterized by the area under the receiver operating characteristic curve (AUC) of the out‐of‐sample predictions.

## 3. Results

Sample demographics are listed in Table 1. Due to the noise floor of the HL2, Traditional‐MC trials with Sway areas < 1 cm^2^ were excluded from the analyses (0.6% of all trials). Twenty‐nine (9%) trials experienced technical errors during data collection or postprocessing and were removed from the analyses. The distribution was well‐balanced between postural control stances with technical errors observed in 7.5%–12% of all trials per stance. Technical error rate is consistent with published works employing MMC [24].

**TABLE 1:** Participant demographics.

	HC (*N* = 35)	PD (*N* = 72)
Age (yrs)	66.0 (7.6)	68.1 (7.7)
Race		
Asian	1 (2.9%)	0 (0%)
White	34 (97.1%)	67 (93.1%)
Black	0 (0%)	5 (6.9%)
Gender		
Female	20 (57.1%)	17 (23.6%)
Male	15 (42.9%)	55 (76.4%)
Education (yrs)	17.7 (3.6)	16.8 (2.3)
Years since PD diagnosis	—	6.2 (4.4)
MDS‐UPDRS III motor score (off‐meds)	—	36.7 (14.6)
Severity score (off‐meds)		
Mild	—	29 (40.3%)
Moderate	—	36 (50.0%)
Severe	—	7 (9.7%)

*Note:* Data presented as mean (SD) or *n* (%).

### 3.1. CART‐MMC Outcomes Demonstrate Criterion Validity With Traditional‐MC

For the subset of participants who completed the Traditional‐MC data collection (54 total; PD = 26; HC = 28) and completed the 30‐s stance, equivalence was observed between CART‐MMC and Traditional‐MC for Sway area and ML range (Table 2). There was not a significant interaction between the stance and system for Sway area, ML range, and AP range (*p* = 0.14, *p* = 0.94, and *p* = 0.06, respectively), and as such, the equivalence of systems was assessed simultaneously across all stances. The magnitude of differences between Traditional‐MC and CART‐MMC was 5% or less for all three stances for Sway area (*p* < 0.01) and 3.2% or less for ML range (*p* < 0.05). The magnitude of differences between Traditional‐MC and CART‐MMC for AP range ranged between 2.3%–8.6% between stances (*p* = 0.34) with Tandem‐Firm‐EO showing the largest difference between systems (8.6%).

**TABLE 2:** CART‐MMC equivalent to Traditional‐MC.

Outcome	Trial type	*n*	Traditional‐MC	CART‐MMC	Percent differences (%)	*p* value
Sway area (cm^2^)	FT‐Firm‐EC	45	5.34 (4.33, 6.59)	5.43 (4.45, 6.63)	1.7	< 0.01^∗^
	Tandem‐Firm‐EO	29	4.02 (3.21, 5.04)	4.22 (3.39, 5.26)	5.0	
	FT‐Foam‐EC	33	16.5 (13.8, 19.9)	16.3 (13.5, 19.5)	−1.8	

ML range (cm)	FT‐Firm‐EC	45	2.05 (1.83, 2.29)	1.99 (1.80, 2.21)	−2.5	< 0.05^∗^
	Tandem‐Firm‐EO	29	2.22 (1.99, 2.48)	2.15 (1.94, 2.39)	−3.2	
	FT‐Foam‐EC	33	4.01 (3.58, 4.48)	3.92 (3.50, 4.38)	−2.2	

AP range (cm)	FT‐Firm‐EC	45	2.25 (2.00, 2.55)	2.33 (2.07, 2.62)	3.3	0.34
	Tandem‐Firm‐EO	29	1.62 (1.34, 1.94)	1.75 (1.47, 2.09)	8.6	
	FT‐Foam‐EC	33	3.59 (3.22, 4.01)	3.67 (3.32, 4.06)	2.3	

*Note:* Data presented as median (95% CI). For each outcome, equivalence was assessed across all completed stances simultaneously when the interaction between the stance and system was not significant; otherwise, equivalence was assessed separately within each stance.

^∗^ *p* < 0.05.

### 3.2. ML Stability was More Impaired in PD Patients Compared to HCs

As measured by the CART‐MMC, the median ML range was 17% greater in PD than in HCs during the FT‐Firm‐EC stance (*p* < 0.05; Table 3) and 16% greater in the Tandem‐Firm‐EO stance (*p* = 0.06). The median AP ranges were 4.8% and 9.2% larger in PD for FT‐firm‐EC and Tandem‐Firm‐EO (*p* = 0.56 and *p* = 0.52, respectively). The median PD Sway areas for FT‐Firm‐EC and Tandem‐Firm‐EO stances were 27% and 26% larger (*p* = 0.06 and *p* = 0.18, respectively). For FT‐Foam‐EC, the magnitude of relative differences between group medians was less than 7.1% for all three metrics, and none were significantly different (both *p* > 0.54). Notably, the completion rates were lower for the PD group across stances when compared to the HC group, effectively removing a larger proportion of PD with poor postural control in the more challenging stances and thus limiting the comparison between the HC and PD groups.

**TABLE 3:** PD demonstrate larger movements across stances.

Outcome	HC, *n*	PD, *n*	HC	PD	Percent difference (%)	Hedges’ g	*p* value
FT‐Firm‐EC							
95% sway area (cm^2^)	31	62	4.87 (4.17, 5.68)	6.16 (5.12, 7.41)	26.5	0.38	0.06
95% ML range (cm)			1.89 (1.71, 2.09)	2.21 (2.02, 2.41)	16.9	0.48	< 0.05^∗^
95% AP range (cm)			2.21 (1.98, 2.46)	2.31 (2.07, 2.58)	4.8	0.12	0.56
Tandem‐Firm‐EO							
95% sway area (cm^2^)	22	33	3.50 (2.72, 4.51)	4.40 (3.58, 5.41)	25.6	0.37	0.18
95% ML range (cm)			1.90 (1.73, 2.10)	2.20 (1.96, 2.48)	15.9	0.50	0.06
95% AP range (cm)			1.61 (1.30, 1.99)	1.76 (1.50, 2.06)	9.2%	0.18	0.52
FT‐Foam‐EC							
95% sway area (cm^2^)	26	46	15.3 (12.4, 18.9)	16.4 (14.4, 18.7)	7.1	0.13	0.59
95% ML range (cm)			3.68 (3.25, 4.16)	3.85 (3.54, 4.18)	4.7	0.15	0.55
95% AP range (cm)			3.59 (3.20, 4.02)	3.55 (3.28, 3.85)	−0.9	−0.03	0.90

*Note:* Data presented as median (95% CI). Hedges’ g is calculated on the transformed scale. The sample includes only completed trials.

^∗^ *p* < 0.05.

### 3.3. Automated LOB Detection Aligns With Clinician Visual Assessment

**FIGURE 2:**
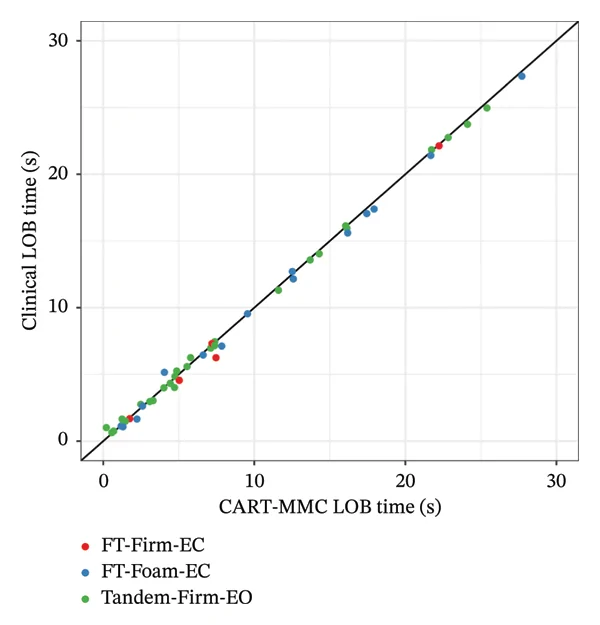
Time to LOB as determined by the CART‐MMC against clinician‐determined time for all trials where both the CART‐MMC and clinician determined an LOB occurred. The CART‐MMC demonstrated LOB detection time equivalent to that of a human rater in addition to high accuracy, sensitivity, and specificity (> 95%) in LOB event detection.

A secondary analysis was performed to investigate the trial completion rate to identify participants who experienced an LOB during each stance. LOB detection for CART‐MMC was automated via a custom algorithm and compared to LOB detection by a clinician’s video review of each trial. Collapsed across stances and groups, the clinician identified an LOB in 52/272 (19%) trials (FT‐Firm‐EC = 5, FT‐Foam‐EC = 16, and Tandem‐Firm‐EO = 31). The CART‐MMC algorithm correctly determined the LOB status (yes or no) in 270 of 272 trials (accuracy = 99.3%). Of the 52 trials where a clinician detected LOB occurred, the CART‐MMC algorithm detected 50 of them (sensitivity = 96.2%). Of the 220 trials without an LOB, the CART‐MMC algorithm correctly identified no LOB in all 220 (specificity = 100%). Across all participants and stances, mean time until LOB was significantly equivalent between the CART‐MMC (8.99 s) and clinician identification (8.88 s) (*p* < 0.001). Figure 2 compares the CART‐MMC and clinician‐identified LOB times, for trials where a LOB occurred.

### 3.4. Postural Control Impairments in PD Represented by Greater Probability of LOB

**FIGURE 3:**
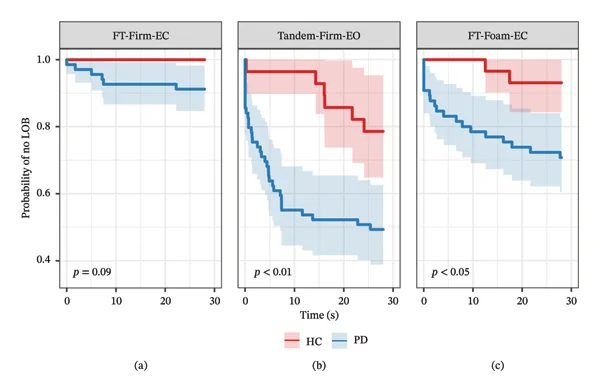
Kaplan–Meier plots of LOB by group over time within each stance. Participants who were unable to initiate the stance were included in the analysis; hence, the reduction in probability of no LOB at the 0 s crossing for all stances. The FT‐Firm‐EC stance (a) demonstrated that the presence of PD did not result in a higher probability of losing balance compared to HCs. Including all performances for each stance highlights the impairment in the PD group’s postural control in the more challenging Tandem‐Firm‐EO (b) and FT‐Foam‐EC (c) stances that could not be quantified during the strict postural control assessment. The shaded region represents 95% confidence intervals. The *p* values were computed from a log‐rank test comparing survival curves.

The proportion of excluded trials was examined per group and stance condition to assess whether the inability to complete the stance reflected random variability or was disproportionately associated with higher task difficultly across each group. Collapsed across stances and groups, 23% of the trials had an LOB. Notably, 51% of the Tandem‐Firm‐EO trials for the PD group resulted in an LOB. For FT‐Firm‐EC, 6 (9%) PD patients experienced an LOB compared to 0 (0%) HC had an LOB, and the probability of not experiencing an LOB was not significantly greater for PD than HCs (*p* = 0.09) (Figure 3a). For FT‐Foam‐EC, 19 (29%) PD patients compared to 2 (7%) HCs experienced an LOB or were unable to start, significantly differentiating PD as being less likely to maintain the stance than HCs (*p* < 0.05) (Figure 3b). For Tandem‐Firm‐EO, 35 (51%) PD patients and 6 (21%) HCs experienced an LOB or were unable to start, significantly differentiating PD performance as being less likely to maintain the stance than HCs (*p* < 0.01) (Figure 3c).

### 3.5. FT‐Firm‐EC Performance Predictive of LOB During Challenging Stances in PD Group

**FIGURE 4:**
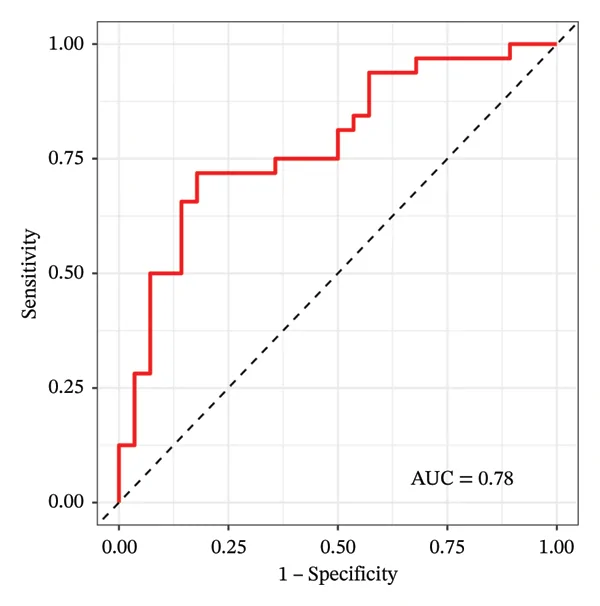
In the relaxed LASSO model developed on FT‐Firm‐EC data, one predictor, ML range, discriminated whether an LOB would occur in PD during the Tandem‐Firm‐EO stance (LOO‐CV AUC = 0.78). Larger ML range values during FT‐Firm‐EC were associated with increased predicted probabilities of an LOB during Tandem‐Firm‐EO.

An exploratory analysis that leveraged the high completion rate of the FT‐Firm‐EC stance in the PD group (91% of PD patients completed) was performed to identify balance metrics during the FT‐Firm‐EC stance that were associated with LOB during the Tandem‐Firm‐EO stance. Using a relaxed LASSO model (Figure 4), one outcome was selected, ML range (odds ratio = 0.136). The LOO‐CV AUC value was 0.78.

## 4. Discussion

The CART‐MMC provides equivalent measures of postural control relative to gold‐standard Traditional‐MC. Data derived from RGB and depth cameras coupled with human pose estimation software provide objective and quantitative data related to static postural control patterns that traditional clinical balance assessments fail to provide. The CART‐MMC platform provides a viable, affordable, and scalable alternative approach to Traditional‐MC to precisely characterize postural control capabilities of PwPD.

Critical to adopting and implementing technology into the clinical workflow is that the resultant data must be accurate and valid to ensure clinicians can trust what the data are communicating about motor function [16]. The recommended path for trusting data from a new technology is to determine if the proposed technology outcomes are equivalent to gold‐standard outcomes that capture the movement or motor control strategy of interest [15]. It is important to note that clinical measures of motor symptoms, while often considered the clinical gold standard, are not gold standards of precise quantification of movement or motor control. To date, the published works on MMC postural control data have not adopted a strict equivalence testing approach in their validation to a biomechanical gold standard [25, 26]. While correlating MMC data to clinical assessments ties the outcomes to a practical and relatable assessment, correlation to a non–gold‐standard assessment does not allow for determining accuracy of a system [27]. Visual inspection of error and bias addressed via Bland–Altman plots provide an understanding of agreement between systems but lack a quantifiable outcome that states the systems are equivalent [28]. Utilizing alternative statistical approaches to mistakenly infer equivalence has potential to disrupt the integration of MMC in clinical practice. Postural control outcomes, Sway area and ML range, from CART‐MMC were within 5% and considered equivalent to Traditional‐MC, thus establishing CART‐MMC as a valid method of acquiring postural control data. Although statistical equivalence was not achieved for the outcome of AP range, differences from Traditional‐MC were between 0.8–1.3 mm, which are comparable to errors previously reported using the Vicon Motion Capture system of 2 mm [29]. In addition, modest overestimation by MMC in the AP direction was also observed by Valenzuela and colleagues [28] relative to the ML direction when tracking COM during static postural control tasks in young adults. Considering the work of Valenzuela et al. in combination with the results from CART‐MMC, submillimeter excursions in the AP direction are difficult to precisely quantify with MMC. Given the task demands of the present study, the challenging stances perturb sensorimotor feedback and elicit larger, more functionally meaningful corrective movements, reducing the clinical relevance of capturing submillimeter excursions. The HC and PD groups in the present study demonstrated movement amplitudes exceeding 1.5 cm, a range in which MMC‐derived measures are more robust and clinically interpretable.

Known‐group validity assessed between the HC and PD groups was completed using only full‐duration trials (i.e., no LOB). This approach minimizes ambiguity in determining when to truncate data when an LOB did occur and how to interpret data from vastly different trial durations in which the participant was meeting the requirements of the stance. In the easiest balance stance, FT‐Firm‐EC, only 8% of the PD group experienced an LOB and 0% of the HC group experienced an LOB. For this condition, a significantly larger ML excursion was present in the PD group and is consistent with previous work [30]. It is well‐documented that ML movement is more pronounced in PwPD compared to HCs, appears earlier in the disease course, and serves as a more sensitive marker of postural control impairments than sway area, thus explaining the usefulness in serving as the sole predictor in the LOB prediction model [7]. The Sway areas and AP ranges did not differ between groups, which previous works indicate varied results of sway area when compared to HCs [31–33]. Studies that stratified PD cohorts by the presence of freezing of gait and likely greater postural instability and gait difficulty, evidence for impaired multiplanar postural control remains inconsistent and appears to be highly task‐dependent [34]. The PD group performance relative to the HC group observed in this study is likely not reflective of preserved postural control, but rather a consequence of methodological constraints. Specifically, individuals who would have demonstrated larger sway were systematically excluded due to LOB, effectively truncating the upper end of postural instability within the PD group. This exclusion reduces variability and biases the sample toward higher‐functioning individuals, thereby masking true group differences. The resultant reduction in sample size, most notably within the PD group, further emphasizes the difficulty of obtaining full‐duration postural control trials in this population and highlights a critical limitation in balance assessment paradigms that rely on trial completion.

While averaging performance of several trials is common in postural control research, a single trial was intentionally used to minimize trial‐dependent habituation and strategy optimization, which are known to occur with repeated exposure to balance tasks, including in PD [9]. Repeated trials can lead to reduced sway through short‐term adaptation, potentially obscuring underlying postural instability [35]. The stances selected for the study imposed substantial sensory and biomechanical constraints such that the initial trial is likely to best reflect unacclimated, ecologically valid postural control.

To better understand postural control of the ecological data, the survival analysis was conducted to determine the effect of stance on time to LOB between groups and to leverage the CART‐MMC automated LOB detection algorithm. The FT‐Firm‐EC stance observed the same statistical probability of no LOB between groups. As the postural control stances progressed in difficulty, there was a separation in the groups with the PD group demonstrating a higher probability of LOB compared to HCs. The addition of a perturbed stance surface in the FT‐Foam‐EC condition appears to have exacerbated known deficits in sensory integration in the PD group, which aligns with prior work demonstrating maladaptive proprioceptive weighting on unstable surfaces in PD [36, 37]. Notably, despite the restoration of visual input, the Tandem‐Firm‐EO condition elicited the highest likelihood of LOB in the PD group. This condition constrained the frontal plane base of support, an axis in which individuals with PD are especially unstable [7] and resulted in over 85% of PD LOB trials terminating within 10 s, compared to none in healthy controls. Collectively, these findings suggest that reduced frontal plane stability, rather than sensory deprivation alone (i.e., vision occlusion), drives rapid balance failure in PD patients and may reflect impaired or insufficient corrective postural strategies. Integrating the known‐group comparisons with survival analyses yields a more comprehensive understanding of postural control. Full trial duration performance used in the known‐group analysis captures corrective postural adjustments that remain within an individual’s limits of stability; however, interpreting reduced sway as inherently optimal can be misleading. In some PwPD, truncal stiffening due to axial rigidity may artificially constrain COM movements, resulting in smaller sway areas than those observed in HCs [38]. Incorporating time to LOB complements these findings by revealing latent deficits in postural control. Specifically, impairments in both sensorimotor feedback mechanisms and anticipatory (feedforward) control may disproportionately affect the PD group yet remain undetected by traditional clinical assessments or analyses that are limited to successfully completed trials.

The ability to predict LOB during postural control tasks advances the understanding of postural control deficits in PwPD. Higher values of ML range during the FT‐Firm‐EC stance are predictive of LOBs in Tandem‐Firm‐EO, indicating an impairment in ML stability that is not overcome by visual feedback. When ML range is larger in a less challenging FT‐Firm‐EC stance, it is likely the individual is at or near their postural control capacity and that failure is imminent when tasked with a more challenging stance. Equipping clinical and rehabilitative providers with objective postural control metrics that are directly linked to LOB outcomes can facilitate the development of individualized intervention strategies, targeted activity modifications, and patient‐specific education. Such an approach moves beyond generalized, one‐size‐fits‐all models of rehabilitation care and supports precision‐based balance management. Demonstrating predictive LOB capabilities from CART‐MMC positions the technology to be used safely at home as 93% (67/72) of the PD patients were able to complete the trial. Leveraging the predictive capacity of a single, information‐rich stance to eliminate redundant testing conditions enables a more efficient at‐home assessment paradigm. This approach not only preserves the ability to extract meaningful biomechanical data but also creates the opportunity to prioritize the collection of additional motor outcomes, which are essential for a more comprehensive characterization of functional impairment [11, 12, 39]. Prediction models centered on LOB events during stance tasks provide a direct and clinically meaningful pathway for fall risk stratification. Because LOB reflects a breakdown in postural control under controlled conditions, its occurrence serves as a mechanistic proxy for susceptibility to real‐world falls. Prior work has demonstrated the utility of machine learning approaches applied to postural control data for fall risk prediction, though these efforts have largely relied on force plate‐derived metrics [40]. In this context, CART‐MMC extends this framework by enabling LOB‐based prediction from a single, informative stance using a lightweight, portable system. This approach not only preserves predictive validity but also enhances feasibility for at‐home deployment, where rapid risk stratification could be used to proactively initiate targeted rehabilitative interventions.

## 5. Conclusions

The CART‐MMC platform provides equivalent postural control outcomes to Traditional‐MC utilizing a single device that houses RGB and depth cameras in healthy older adults and PwPD. While earlier publications note shortcomings in MMC accurately measuring static postural control, our data suggest the contrary, but it is advisable to validate each MMC system following the equivalence analysis approach. Additionally, objective metrics derived from CART‐MMC were leveraged to predict LOB in a separate, more challenging stance condition. With a growing interest in remote monitoring and assessments, measuring postural control in a manner that is safe but still sensitive to impairments can facilitate efficient remote motor function testing paradigms with MMC. Since ML range provided a good prediction of LOB on a more challenging postural control stance, future studies are being contemplated to leverage the CART‐MMC to predict falls.

### 5.1. Limitations

As is common, the present sample was weighted towards males with PD. Recent meta‐analysis and systematic review points to the overall prevalence ratio of males:females diagnosed with PD at 1.18 [41]. Future studies are required to determine whether females exhibit the same postural control responses as their male counterparts. For PwPD, data were collected in the off‐medication state, which may not accurately depict their postural control responses during their typical waking hours of the day when they are motorically optimized through use of anti‐Parkinsonian medications. Anti‐Parkinsonian medications can increase sway in some PwPD [42]; however, data related to how medication status affects perturbation responses are inconsistent [43, 44].

## Author Contributions

Conceptualization: Jay L. Alberts, Mandy Miller Koop, and Anson B. Rosenfeldt; methodology: Andrew Bazyk, Jay L. Alberts, Mandy Miller Koop, Anson B. Rosenfeldt, Ryan D. Kaya, Colin Waltz, and Joshua D. Johnston; software: Andrew Bazyk; validation: Andrew Bazyk, Eric Zimmerman, and Ryan D. Kaya; formal analysis: Andrew Bazyk, Eric Zimmerman, and Ryan D. Kaya; investigation: Colin Waltz and Ryan D. Kaya; resources: Jay L. Alberts; data curation: Andrew Bazyk, Eric Zimmerman, and Ryan D. Kaya; writing–original draft preparation: Ryan D. Kaya; writing–review and editing: Andrew Bazyk, Eric Zimmerman, Joshua D. Johnston, Benjamin L. Walter, Adam Margolius, Anson B. Rosenfeldt, Mandy Miller Koop, and Jay L. Alberts; visualization: Andrew Bazyk, Eric Zimmerman, and Ryan D. Kaya; supervision: Jay L. Alberts; project administration: Colin Waltz and Ryan D. Kaya; and funding acquisition: Jay L. Alberts and Mandy Miller Koop.

## Funding

This work was supported by a grant from the National Institutes of Health (Grant No. 5R01NS129115‐04) and Edward and Barbara Bell Family Chair. Participant recruitment was aided by funding from the Clinical and Translational Science Collaborative of Northern Ohio, which is funded by the National Institutes of Health, and the National Center for Advancing Translational Sciences, Clinical and Translational Science Award grant, No. UM1TR004528.

## Disclosure

The funders had no role in the design of the study; in the collection, analyses, or interpretation of data; in the writing of the manuscript; or in the decision to publish the results. All authors reviewed and approved the final manuscript.

## Ethics Statement

This study was reviewed and approved by the Cleveland Clinic Institutional Review Board (IRB # 23‐1146). All participants provided written informed consent before inclusion in the study.

## Conflicts of Interest

Jay L. Alberts, Mandy Miller Koop, and Anson B. Rosenfeldt have authored intellectual property associated with the augmented reality assessment modules that have been licensed to a commercial entity. The other authors declare no conflicts of interest.

## Data Availability

The data that support the findings of this study are available from the corresponding author upon reasonable request.
